# Morphology Controlled Synthesis of Composition Related Plasmonic CuCdS Alloy Nanocrystals

**DOI:** 10.3389/fchem.2020.628536

**Published:** 2020-12-23

**Authors:** Yan Gao, Lei Wang, Guimin Tian, Shuaipu Zang, Hongzhe Wang, Jinzhong Niu, Lin Song Li

**Affiliations:** ^1^Key Lab for Special Functional Materials, Ministry of Education, National and Local Joint Engineering Research Center for High-Efficiency Display and Lighting Technology, Collaborative Innovation Center of Nano Functional Materials and Applications, School of Materials Science and Engineering, Henan University, Kaifeng, China; ^2^College of Materials Engineering, Henan University of Engineering, Zhengzhou, China

**Keywords:** morphology, composition, plasmonic, CuCdS, alloy nanocrystals

## Abstract

Cu-based ternary alloy nanocrystals have emerged for extensive applications in solar cells, light-emitting devices (LEDs), and photoelectric detectors because of their low-toxicity, tunable band gaps, and large absorption coefficients. It is still an enormous challenge that regulating optical and electrical properties through changing their compositions and shapes in alloy nanocrystals. Herein, we present a facile method to synthesize CuCdS alloy nanocrystals (NCs) with tunable compositions and shapes at relatively low temperature. Different morphologies of monodisperse CuCdS nanocrystals are tailored successfully by simply adjusting the reaction temperature and Cu:Cd precursor molar ratio. The as-synthesized nanocrystals are of homogeneous alloy structures with uniform obvious lattice fringes throughout the whole particles rather than heterojunction structures. The localized surface plasmon resonance (LSPR) absorption peaks of CuCdS NCs are clearly observed and can be precisely tuned by varying the Cu:Cd molar ratio. Moreover, current–voltage (*I–V*) behaviors of different shaped CuCdS nanocrystals show certain rectification characteristics. The alloy CuCdS NCs with tunable shape, band gap, and compositionpossess a potential application in optoelectronic devices.

## Introduction

Cu-based ternary semiconductor nanocrystals are of great scientific interest for applications in solar cells (Rivest et al., 2011), light-emitting devices (LEDs) (Yoon et al., 2019; Li et al., 2020), biological imaging (Meng et al., 2017), photo-catalytic (Zhao et al., 2016), photoelectric detectors because of their low-toxicity, tunable band gaps, and high absorption coefficients (Pejova et al., 2020). Especially, ternary nanocrystals based on Cu_1.94_S, with localized surface plasmon resonance due to the large number of copper vacancies, have been widely demonstrated for photoelectrochemical water reduction and solar cells due to their high concentration of free charge carriers (Liu et al., 2017; Bai et al., 2020; Bera et al., 2020; Zhu et al., 2020). Developing the size-, shape-, and composition-controlled composite nanocrystals is a significant strategy to regulate their energy level position, carrier concentration, and photoluminescence quantum yield for the improvement of device performance.

Semiconductor–semiconductor hetero-nanostructures have been widely synthesized by hot-injection method and seed growth method (Yi et al., 2010; Shen H. et al., 2012; Li et al., 2013, 2014a). Antecedently, researches on transition-metal and II-VI composite nanocrystals mainly focused on semiconductor–semiconductor heterostructures such as Ag_2_S-ZnS, Ag_2_S-CdS, Ag_2_Se-ZnS, CuS-ZnS, Cu_1.94_S-CdS, Cu_1.94_S-Zn_x_Cd_1−x_S, which have been applied in related optoelectronic devices (Regulacio et al., 2011; Shen et al., 2011a; Zhang et al., 2011; Han et al., 2012; Xu et al., 2013; Wang et al., 2014). And the Cu_1−x_S_x_-CdS has been particularly investigated by researchers. For example, it has been reported in 1980 that the design and fabrication of thin-film CdS/Cu_2_S heterojunction solar cells with energy conversion efficiencies in sunlight of up to 9.15% (Devaney et al., 1979). Wang et al. employed n-CdS/p-Cu_2_S coaxial nanowire to fabricate photovoltaic devices (Pan et al., 2012). However, alloyed composite semiconductors of transition-metal and II-VI elements are extremely rare. It tends to evolve into heterostructures and is difficult to form alloyed nanostructures (Regulacio et al., 2011; Shen et al., 2011a; Wang et al., 2014; Xu et al., 2020). However, alloy nanomaterials have shown excellent characteristics, for instance, alloy quantum dots of II-VI and I-III-VI group show exceptional photovoltaic properties and stability of fluorescence (Swafford et al., 2006; Jun and Jang, 2012; Panthani et al., 2013; Feng et al., 2020). Recently, Cu-based ternary nanostructures such as Cu_3_BiS_3_ and CuMS_2_ NCs (M: In and Ga) were prepared through a cation exchange process. Interestingly, these ternary nanocrystals possessed the alloyed nanostructure instead of a heterostructure. And they revealed enhanced photocatalytic activities toward photoelectrochemical water reduction, owing to their tailored optical band gaps, energy level alignments, and shape affecting (Bera et al., 2020; Zhu et al., 2020). Therefore, further exploiting new methods to synthesize highly crystallized transition-metal and II-VI alloyed nanostructures still worth systematical study.

Herein we report the formation of CuCdS ternary alloy semiconductors *via* a facile one-pot colloidal route. CuCdS nanocrystals with adjustable shapes such as fusiform, rod, hexagon, and rhombus were synthesized by heating CuI and Cadmium diethyldithiocarbamate (Cd(DDTC)_2_, DDTC=diethyldithiocarbamate) as precursors at low temperature. These shapes were tuned by controlling the reaction temperature and Cu:Cd precursor molar ratio. High resolution transmission electron microscopy (HRTEM) and X-ray diffraction (XRD) results indicated the homogeneous alloy structure and high crystallinity. The localized surface plasmon resonance (LSPR) absorption spectrum and current–voltage *(I–V*) behaviors of different shaped CuCdS nanocrystals have also been studied.

## Materials and Methods

### Materials

Copper iodide (CuI, 98%), Sodium N-diethyldithiocarbamate [Na(DDTC), 99%], and Cd(NO_3_)_2_•4H_2_O (95%) were purchased from XiYa Reagent Company (P. R. China). Oleylamine (OAM, 70%) was purchased from Sigma-Aldrich. Hexanes (analytical grade) and acetone (analytical grade) were purchased from Tianjin Chemical Reagents Co. Ltd. All chemicals were used as received without any further purification.

### Synthesis of Cd(DDTC)_2_ Precursors

The preparation of Cd(DDTC)_2_ was referred to a previously published procedure (Shen S. et al., 2012). Typically, Na(DDTC) (18.02 g, 80 mmol) and Cd(NO_3_)_2_•4H_2_O (12.34 g, 40 mmol) were, respectively, dissolved in 200 mL of deionized water by ultrasonic. Subsequently, Cd(NO_3_)_2_ solution was dropwise added into Na(DDTC) solution and kept stirring for 30 min. The resulting white precipitate was generated immediately when the reaction started. Then, Cd(DDTC)_2_ was formed and washed for several times by deionized water and ethanol, and finally was dried in air at room temperature.

### Synthesis of CuCdS

In a typical procedure, CuI (0.4 mmol) and Cd(DDTC)_2_ (0.1 mmol) were mixed with 8 mL of OAM in a 25 mL three-necked flask at room temperature. The mixture was heated to 120°C at a rate of 15°C/min under magnetic stirring and nitrogen flow for 10 min, and then cooled to room temperature. The brown product was collected by centrifugation (4,000 rpm, 10 min), and washed several times by acetone for further characterization. By changing the molar ratio of Cu/Cd and reaction temperature, different shaped nanostructures could be obtained.

### Device Fabrication

A layered structure of Al/CuCdS/ITO was fabricated for conductivity test. Fixed amount of CuCdS nanocrystals solution (30 mg/mL, dispersed in hexanes) was applied directly to pre-cleaned ITO-coated glass substrates (≤40 Ω/sq.) by spin-casting. And uniform nanocrystal thin films were formed. Finally, Al electrodes were deposited by RF magneton sputtering through a metal mask to form a sandwich structured device.

### Measurements and Characterization

Thermal gravimetric analysis (TGA) was obtained by a TG/DTA 6200 thermogravimetric analyzer (SII Inc.) under nitrogen flow (200 cm^3^/min) with a heating rate of 10°C/min. Transmission electron microscopy (TEM) and high resolution transmission electron microscopy (HRTEM) images were taken by a JEOL JEM-2010 microscope with an accelerating voltage of 200 kV. X-Ray diffraction (XRD) results of nanocrystals were obtained employing an X-ray diffractometer (Philips X' Pert Pro) using Cu-Kα radiation (λ = 1.54 Å) with a step size of 0.02° and a scan rate of 4°/min at a voltage/current of 40 kV/40 mA. Energy-dispersive X-ray spectroscopy data (EDS) was presented on an FEI Quanta 200 scanning electron microscope equipped with a Thermo NORAN System SIX energy dispersive X-ray spectrometer. UV–vis absorption was gained using UV–vis spectroscopy (PE Lambda 950). I–V measurements were acquired using Keithley 4200 source meter.

## Results and Discussion

**Figure 1:**
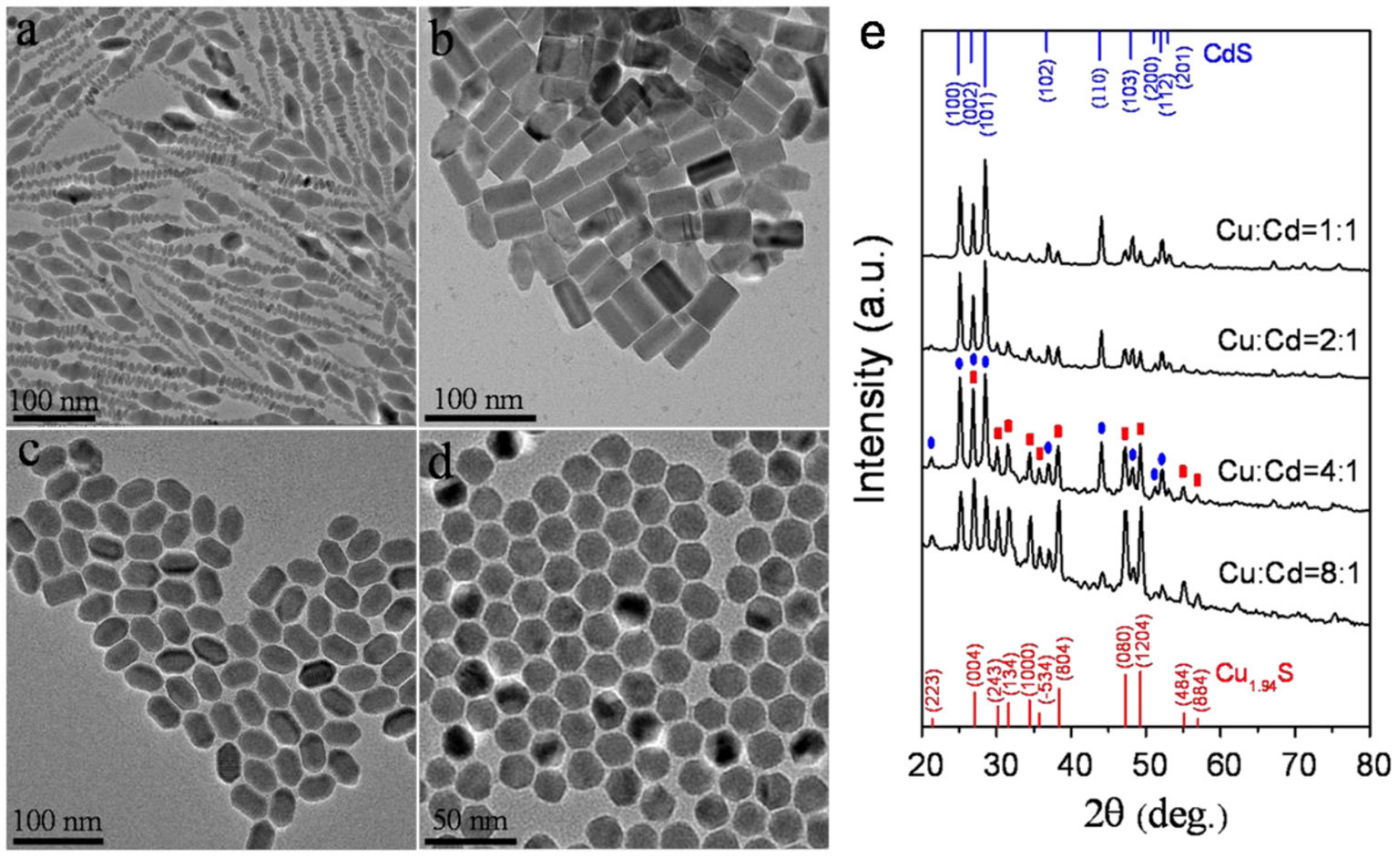
TEM images of CuCdS nanostructures prepared at 120°C with reaction time of 10 min using different Cu/Cd molar ratios: **(a)** 1:1, **(b)** 2:1, **(c)** 4:1, and **(d)** 8:1. **(e)** XRD patterns of the corresponding nanocrystals.

In this report, CuI and Cd(DDTC)_2_ which acted as the reactants were directly dispersed in oleylamine. Among them, Cd(DDTC)_2_ as single-source precursor can simultaneously provide sulfur source and cadmium source (Supplementary Figure 1). The as-prepared Cd(DDTC)_2_ was air-stable and low-toxic. The TGA results illustrated that the decomposition temperature of Cd(DDTC)_2_ in its pure solid form was between about 250 and 350°C in the nitrogen gas environment (Supplementary Figure 2). Owing to the presence of oleylamine, various morphologies of CuCdS alloy nanostructures were synthesized at relatively low temperature. The influence of the reaction conditions on the architectures of CuCdS nanocrystals was mainly researched by changing molar ratio of Cu:Cd and reaction temperature. TEM was used to observe the morphology of as-prepared CuCdS nanocrystals. Figure 1 shows four different shapes of CuCdS alloyed nanostructures, which were obtained by changing the molar ratio of Cu:Cd at 120°C with reaction time of 10 min. CuCdS crystals, which possess different surface energies in different directions, allow the shape of the nanoparticles to be controlled by increasing and/or decreasing the growth rate in different directions (Han et al., 2008). We changed the molar ratio of Cu:Cd from 1:1 to 8:1 and kept the other parameters the same. Low Cu ratio (Cu:Cd = 1:1) leaded to the formation of fusiform morphology, as shown in Figure 1a. However, further changing the Cu:Cd ratio to 2:1 leaded to the formation of rod shape morphology with average length of 44 nm and width of 24 nm (Figure 1b). Contrastively, when the Cu/Cd ratios were increased to 4:1 and 8:1, rod-shaped hexagon and regular hexagonal nanocrystals were finally obtained, respectively (Figures 1c,d).

From the XRD results (Figure 1e), we can find that the diffraction patterns of as-synthesized CuCdS nanocrystals could be regarded as the combination of diffraction patterns of monoclinic Cu_1.94_S (JCPDS No. 23-0959) and wurtzite CdS (JCPDS No. 80-0006). With the increase of Cu content in the reaction, the intensity of Cu_1.94_S peak gradually increased and the intensity of CdS gradually decreased. The evolution of XRD patterns showed that different composed CuCdS nanocrystallines were successfully synthesized without impurities.

**Figure 2:**
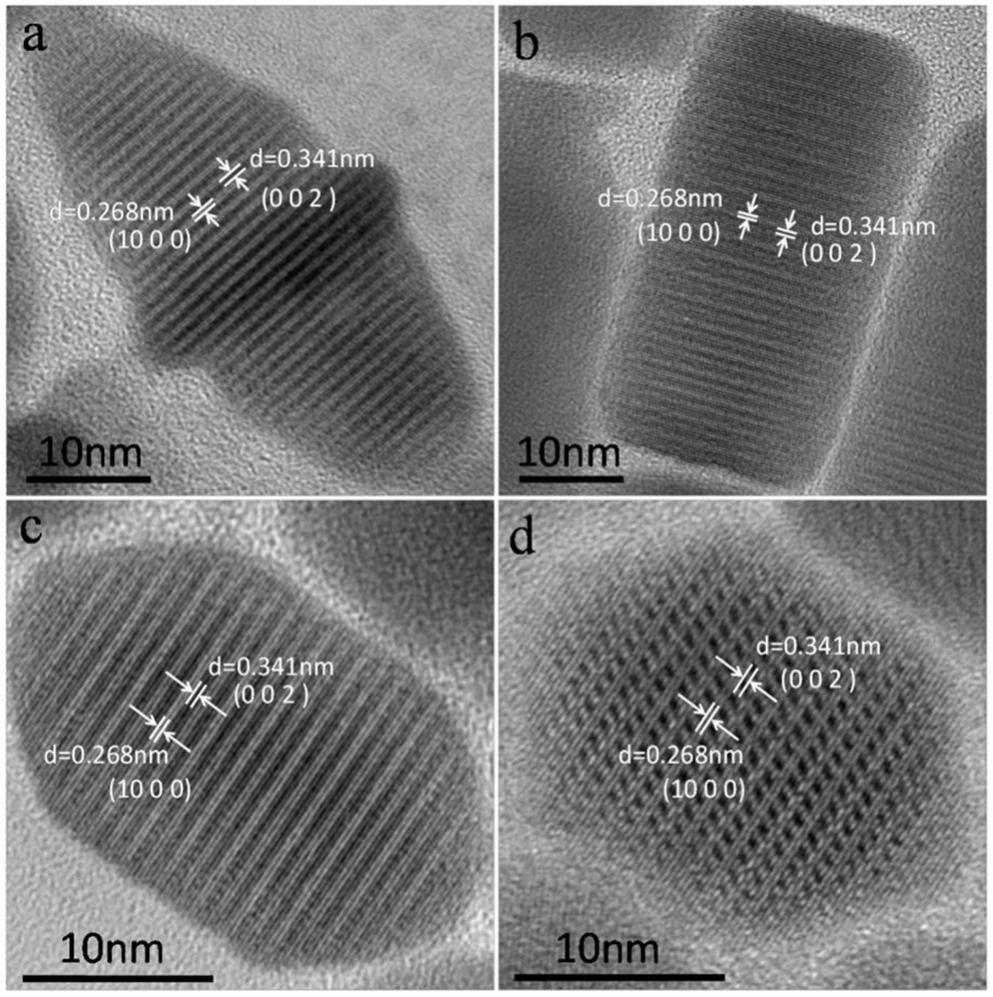
HRTEM images of CuCdS nanostructures prepared at 120°C with reaction time of 10 min using different Cu/Cd molar ratios: **(a)** 1:1, **(b)** 2:1, **(c)** 4:1, and **(d)** 8:1.

The corresponding high-resolution TEM (HRTEM) images of CuCdS nanocrystals synthesized at 120°C with reaction time of 10 min using different Cu/Cd molar ratios are shown in Figure 2. All the HRTEM images of CuCdS nanocrystals show good crystallinity with uniform lattice fringes throughout the whole particle, indicating the homogeneous CuCdS alloy structures, rather than the Cu_1.94_S-CdS heterostructure. The analysis of the different light and dark lattice spacings reveals d_light_ = 0.268 nm and d_dark_ = 0.341 nm, which fit the interplanar spacing of monoclinic Cu_1.94_S (1000) and wurtzite CdS (002) planes, respectively. EDS analysis in Table 1 quantifies the elemental compositions of CuCdS alloy nanocrystals. Cu, Cd, and S signals are all detected, indicating that the alloy nanostructures are composed mainly of Cu, Cd, and S. All the molar ratio of Cu:Cd in products were greater than that of raw materials, declaring that the activity of Cu was higher than that of Cd.

**Table 1:** Elemental percentage of CuCdS nanocrystals with different Cu:Cd molar ratios from EDS results.

**Area**	**1:1**	**2:1**	**4:1**	**8:1**
Cu (At%)	41.11	49.48	63.53	68.38
Cd (At%)	30.50	22.25	05.20	01.74
S (At%)	28.39	28.27	31.26	29.88

**Figure 3:**
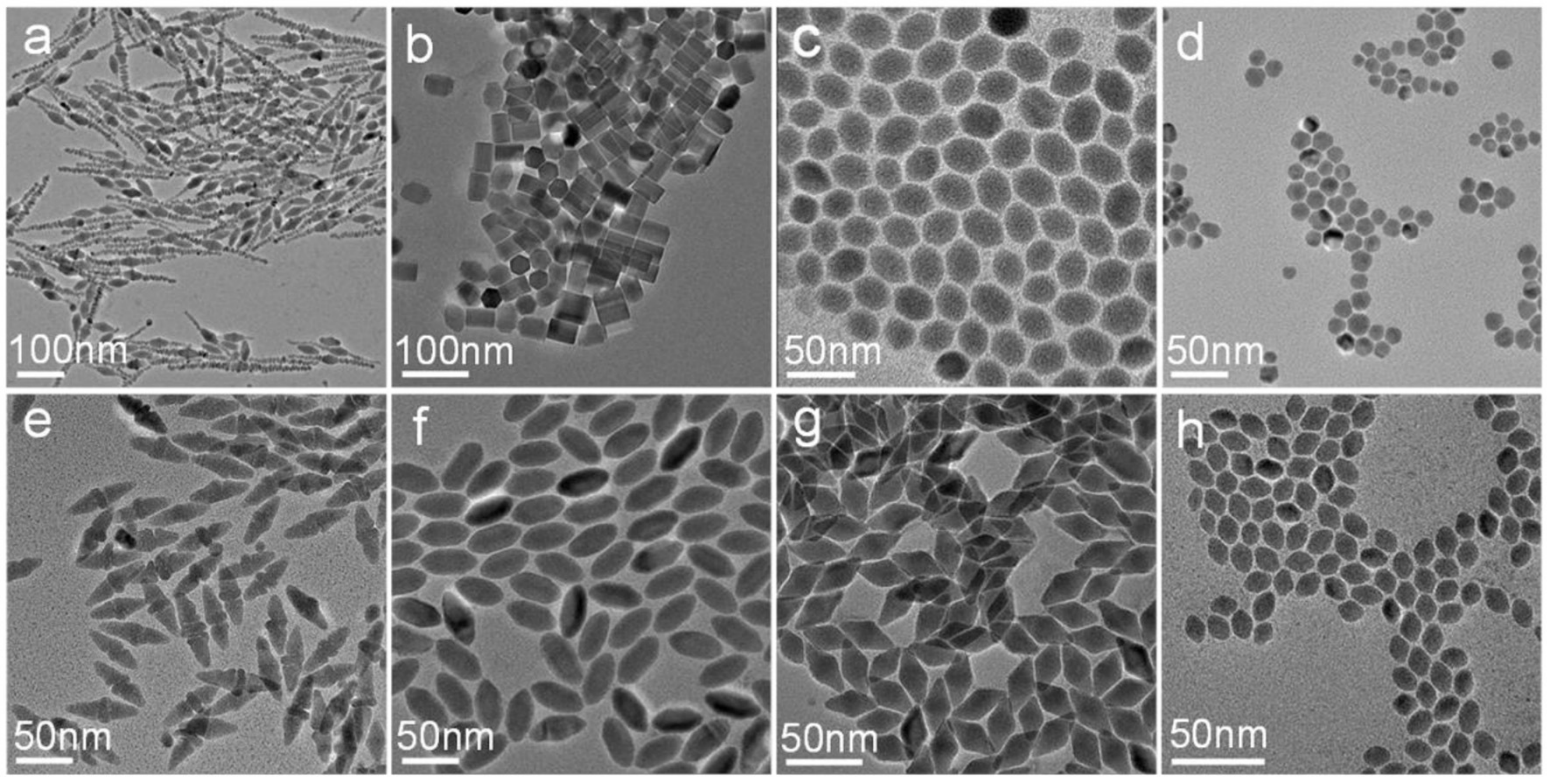
TEM images of the samples prepared by using different temperature and molar ratio of Cu/Cd: **(a)** 100°C, 1:1; **(b)** 100°C, 2:1; **(c)** 100°C, 4:1; **(d)** 100°C, 8:1; **(e)** 150°C, 1:1; **(f)** 150°C, 2:1; **(g)** 150°C, 4:1; **(h)** 150°C, 8:1.

Besides the molar ratio of the reactants, we found that the reaction temperature could also significantly influence the reaction kinetics and the morphology of the obtained CuCdS nanocrystals. For example, when the molar ratio of Cu:Cd was kept of 1:1, and changing the reaction temperature to 100 and 150°C (Figures 3a,e), the shape of CuCdS ternary semiconductors remained fusiform with different length. At the 2:1 Cu/Cd molar ratio, with the reaction temperature decreased to 100°C, CuCdS nanocrystals had some agglomeration with inhomogeneous rectangular and hexagonal morphology (Figure 3b); while, uniform elliptical CuCdS materials were synthesized at 150°C (Figure 3f). The nanocrystals synthesized with Cu:Cd = 4:1 showed rod-shaped hexagon without obvious corner angle at 100°C and unified rhombus at 150°C (Figures 3c,g). For the products with a Cu:Cd molar ratio of 8:1, rising or falling of the temperature similarly caused the change of morphology to nearly rotundity or homogeneous jujube pit (Figures 3d,h). Based on experimental data and literatures. Generally, Cu ions combined with Cd(DDTC)2 forming CuDDTC. Then, the CuDDTC decomposed to obtain Cu_1.94_S crystals with a dominating (100) plane (Han et al., 2012). CdDDTC decomposed to form CdS nanorods with a prominent (002) plane in OAM solution (Shen et al., 2011b). Simultaneously, the evolution of the reaction process includes a cation exchanging between Cu ions and Cd ions to form CuCdS crystals. In summary, the Cu ions dominated the (100) planes of CuCdS crystals and the I^−^ ions were strongly combined with Cu in the precursor. Moreover, Cd ions dominated the (002) planes of CuCdS and were only weakly bonded by the DDTC ligands, the difference of reactivity on different crystal planes resulted in the various shapes. From the results in Figures 1, 3, we found that with the decrease of Cd content at different reaction temperatures, the length to width ratio of CuCdS nanocrystals was reduced.

**Figure 4:**
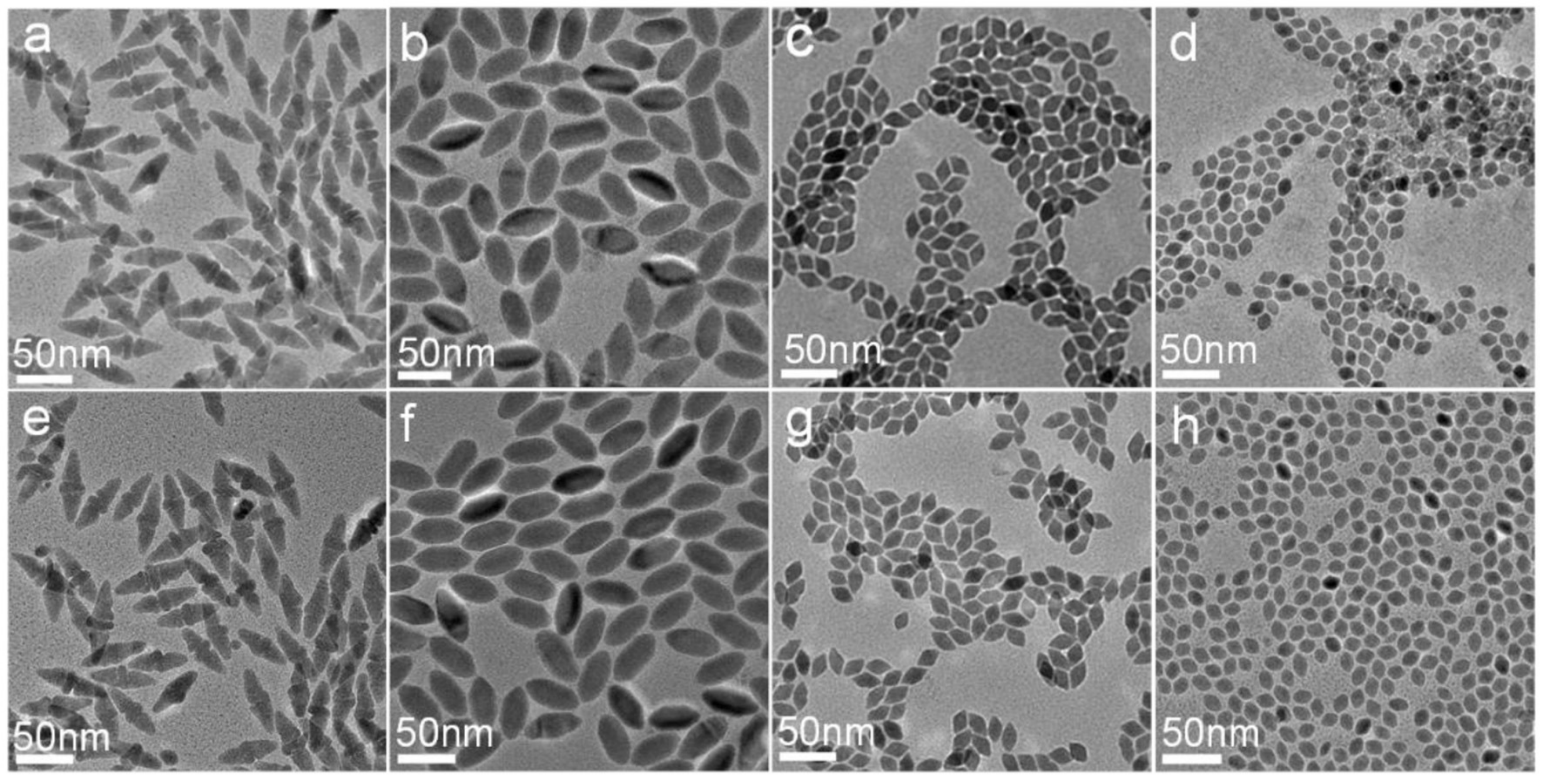
TEM images of the samples prepared by using different molar ratios of Cu/Cd and reaction times at 150°C: **(a)** 1:1, 1 min; **(b)** 2:1, 1 min; **(c)** 4:1, 1 min; **(d)** 8:1, 1 min; **(e)** 1:1, 10 min; **(f)** 2:1, 10 min; **(g)** 4:1, 10 min; **(h)** 8:1, 10 min.

To give an in-depth understanding of the evolution of CuCdS NCs, the effect of reaction time was studied. The size and morphology evolution of the NCs were monitored by TEM observation as shown in Figure 4. Nanocrystals prepared with the reaction time of 1 min and 10 min were almost the same appearance, which indicates that the reaction occurred in the extremely instantaneous time. In the rest of the reaction time, Ostwald ripening dominated the growth process, and the CuCdS nanocrystallines appeared tiny increase in size and tended to be more uniform and dispersive.

**Figure 5:**
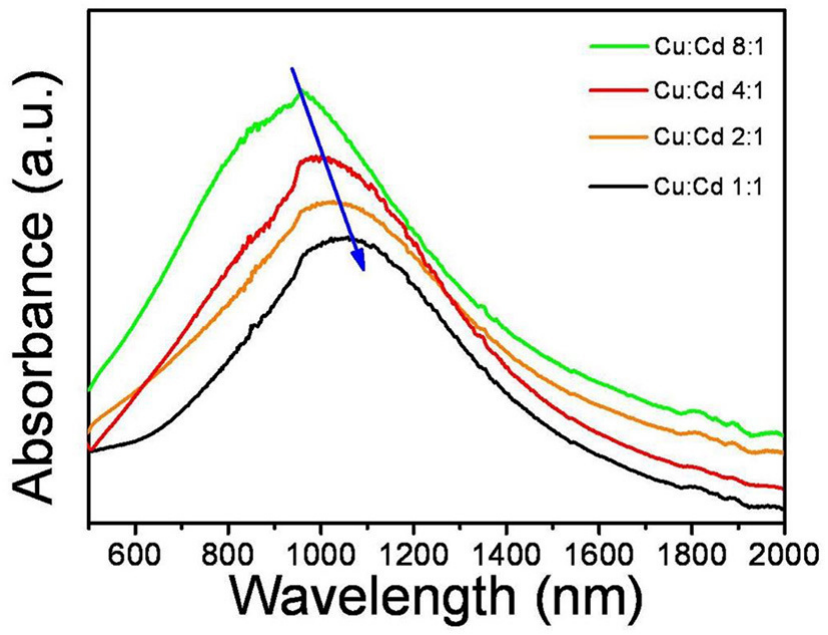
LSPR related absorption spectra of CuCdS nanocrystals synthesized at 120°C with different Cu/Cd molar ratios.

The LSPR related absorption spectra of CuCdS nanostructures with different morphologies by reaction at 120°C are shown in Figure 5. The CuCdS NCs exhibited a NIR-LSPR peak which had a broad band. And the plasmonic absorption band exhibits a red-shift from 960 to 1,067 nm with the decreasing of Cu content in the nanocrystals. The strong LSPR absorption peak was clearly detected at 960 nm for products using 8:1 Cu:Cd molar ratio. With the decreasing of Cu content to Cu:Cd = 4:1, the LSPR absorption peak red-shifted to 1,010 nm. Further decrease of the amount of Cu caused the LSPR peak further red-shifted to 1,043 and 1,067 nm for the NCs prepared using 2:1 and 1:1 Cu:Cd molar ratios. According to previous reports, the LSPR related absorption is dependent on the free carrier density, high frequency dielectric constant, and crystalline anisotropy (Zhou et al., 2016; Zhu et al., 2016). Based on the literatures, the dominant influence of crystalline anisotropy usually results in the adjustable LSPR absorption, which has been demonstrated experimentally and theoretically (Kim et al., 2016). The effect of crystalline anisotropy includes the size, shape and aspect ratio of nanocrystal. The LSPR absorption peak shows a continuous red shift with the decrease of Cu content in these nanocrystals, which is attributed to the decrease of the carrier density (Zhou et al., 2016).

**Figure 6:**
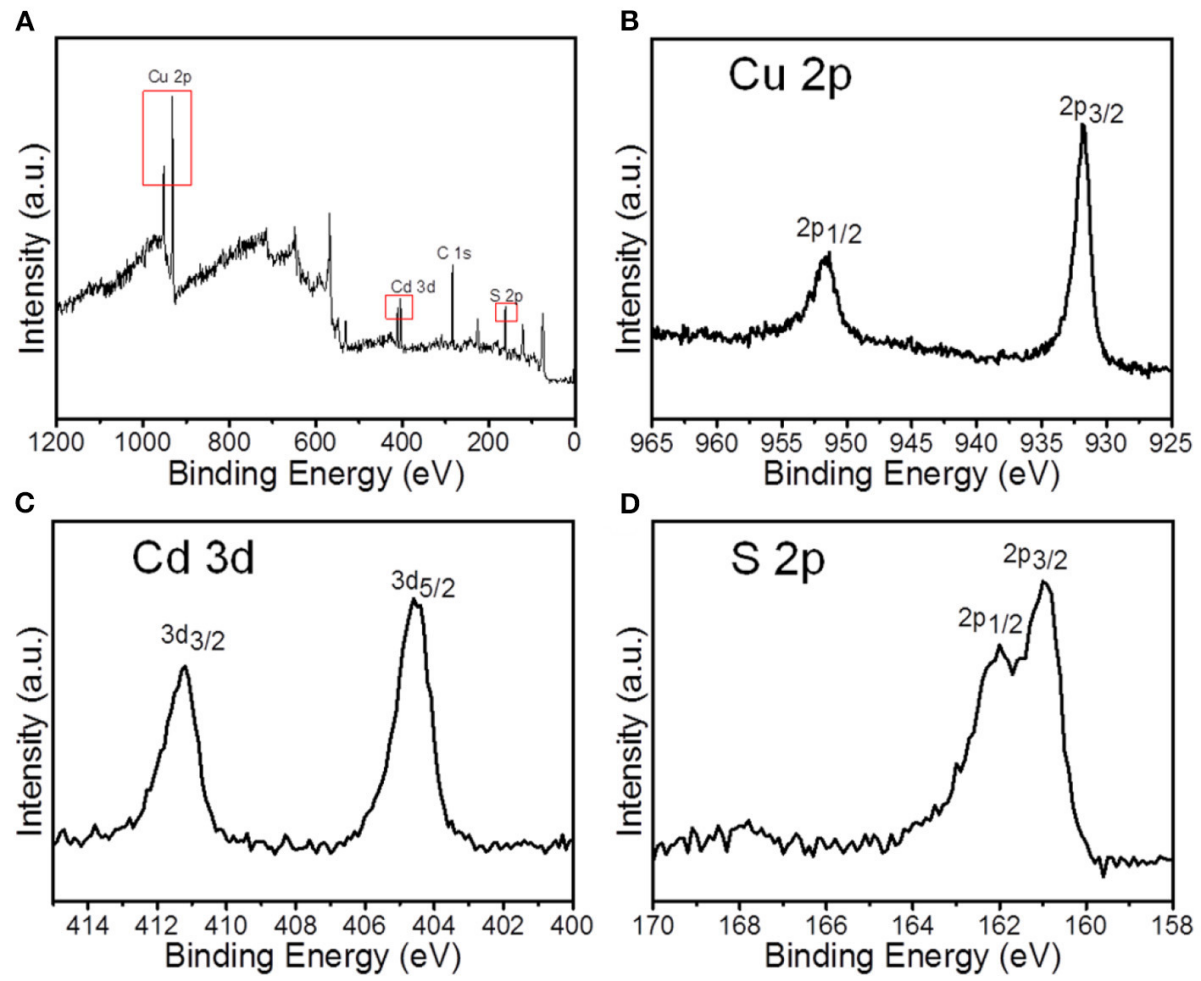
XPS spectra of Cu_1.94_S-CdS nanostructures prepared at 4:1 Cu/Cd molar ratio: **(A)** Survey spectrum; **(B–D)** High-resolution spectra of Cu 2p, Cd 3d, and S 2p core levels, respectively.

The composition and valence states of nanocrystals synthesized with 4:1 Cu/Cd molar ratio and 120°C reaction temperature were further identified by x-ray photoelectron spectroscopy (XPS), as shown in Figure 6. XPS survey spectrum presents the presence of C 1s, Cu 2p, Cd 3d, and S 2p core level peaks. In the high-resolution XPS spectrum of the Cu 2p core level, the 2p3/2 and 2p1/2 signals are, respectively, located at 931.6 eV and 951.1 eV, revealing that the binding energies are well-matched with that of Cu^+^ according to previous reports (Tang et al., 2010). The XPS peaks of Cd 3d is centered at the binding energies of 404.5 eV (Cd 3d5/2) and 411.2 eV (Cd 3d3/2), which are in agreement with the Cd 3d core levels from CdS (Zhu et al., 2014). The featured peaks of S 2p were located at the binding energies of 160.8 eV and 161.9 eV, which indicated the S 2p binding energy with −2 oxidation state on the basis of previous reports (Li et al., 2014b).

**Figure 7:**
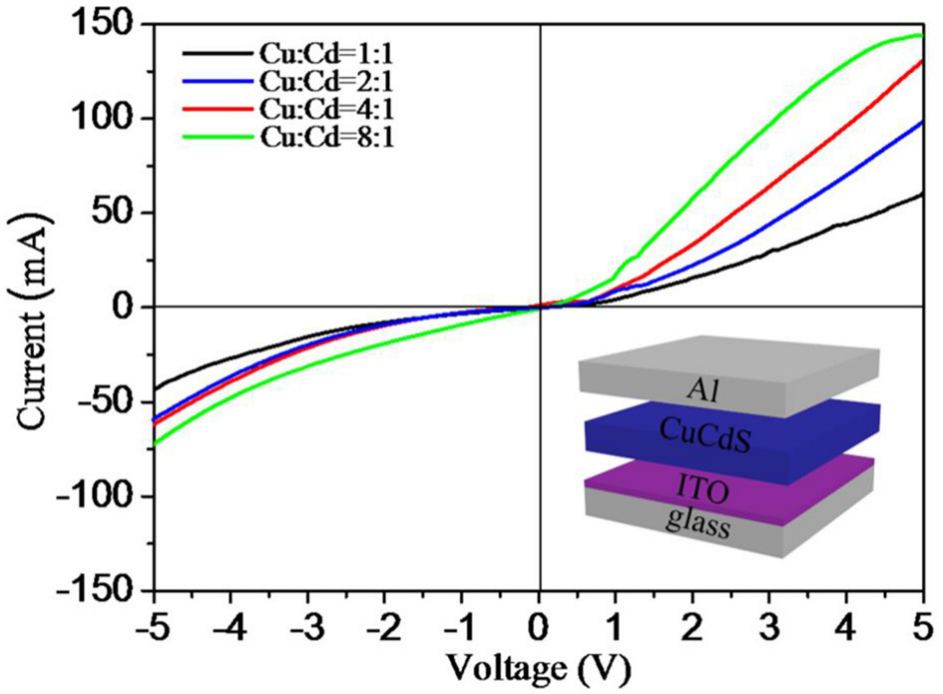
Current-voltage (*I-V*) characteristic curves for CuCdS films prepared by using CuCdS nanocrystals synthesized with different molar ratios of Cu/Cd. Insets: the schematic structure of devices.

The current–voltage (*I–V*) characteristics of four different shaped CuCdS nanocrystals which were synthesized with different Cu:Cd molar ratios are presented in Figure 7. The bottom-right inset of Figure 7 depicts a typical schematic structure of devices, containing ITO anode, CuCdS nanocrystals film, and Al cathode. To measure these devices, we used copper conducting resin to form the contact pads between the probes and the Al electrodes and obtained rectifying *I–V* curves. *I–V* curves of the device were measured for a 5 V bias range at room temperature in air environment. We can gain four asymmetric rectifying *I–V* curves, which running through the positive and the negative bias voltages. For these four different shaped samples, the values of the current show a gradually increasing trend with increasing the molar ratio of Cu:Cd from 1:1 to 8:1. The non-linearity of current illustrates that the nanocrystals formed Schottky junction with Al. Additionally, the change of conductivity may be related to the decrease in the percentage of CdS semiconductor. As a high resistivity semiconductor, CdS may reduce the current values on account of the high series resistance in the diodes. These rectification characteristics implied that the CuCdS alloy nanostructures would has potential occupancy in photovoltaic devices.

## Conclusion

In summary, we report the high-quality CuCdS alloy nanocrystals with tunable compositions and various shapes synthesized by using CuI and Cd(DDTC)_2_ as precursors. The shapes of this products are controlled by reaction temperatures (100–150°C) and Cu:Cd precursor molar ratios, which includes fusiform, rod, hexagon, rhombus and so on. The CuCdS alloy nanocrystals exhibit tunable LSPR absorption spectra dependent on the free carrier density, high frequency dielectric constant, and crystalline anisotropy. In addition, the CuCdS nanocrystals reveal certain rectification characteristics. These interesting properties make the CuCdS alloy nanocrystals applicable in photovoltaic and photoelectric researches.

## Data Availability Statement

The original contributions presented in the study are included in the article/Supplementary Materials, further inquiries can be directed to the corresponding author/s.

## Author Contributions

YG conducted the experiments and wrote the results. LW wrote the manuscript. GT, SZ, and HW characterized the samples. JN and LL revised the manuscript. All authors contributed to the article and approved the submitted version.

## Conflict of Interest

The authors declare that the research was conducted in the absence of any commercial or financial relationships that could be construed as a potential conflict of interest.
