# Supplementary material for: Morphology Controlled Synthesis of Composition Related Plasmonic CuCdS Alloy Nanocrystals
Source: Front Chem. 2020 Dec 23;8:628536. doi: 10.3389/fchem.2020.628536 (PMC7785700; doi:10.3389/fchem.2020.628536)
Supplement: Supplementary file 1 [file Data_Sheet_1.docx]

Supplementary Material

**Morphology Controlled Synthesis of Composition Related Plasmonic CuCdS Alloy Nanocrystals**

Yan Gao,^a^ Lei Wang,^*a^ Guimin Tian,^b^ Shuaipu Zang,^b^ Hongzhe Wang,^a^ Jinzhong Niu,^*b^ Lin Song Li^a^

^a^Key Lab for Special Functional Materials, Ministry of Education, National and Local Joint Engineering Research Center for High-Efficiency Display and Lighting Technology, School of Materials Science and Engineering, and Collaborative Innovation Center of Nano Functional Materials and Applications, Henan University, Kaifeng, 475004, China.

^b^College of Materials Engineering, Henan University of Engineering, Zhengzhou, 451191, China.

***Correspondence:**

Dr. Lei Wang; Jinzhong Niu

E-mail: wanglei7869@henu.edu.cn

E-mail: niujinzhong@gmail.com


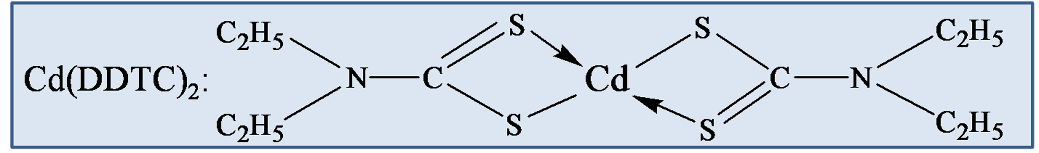


**Figure S1** The chemical structure of Cd(DDTC)_2_.


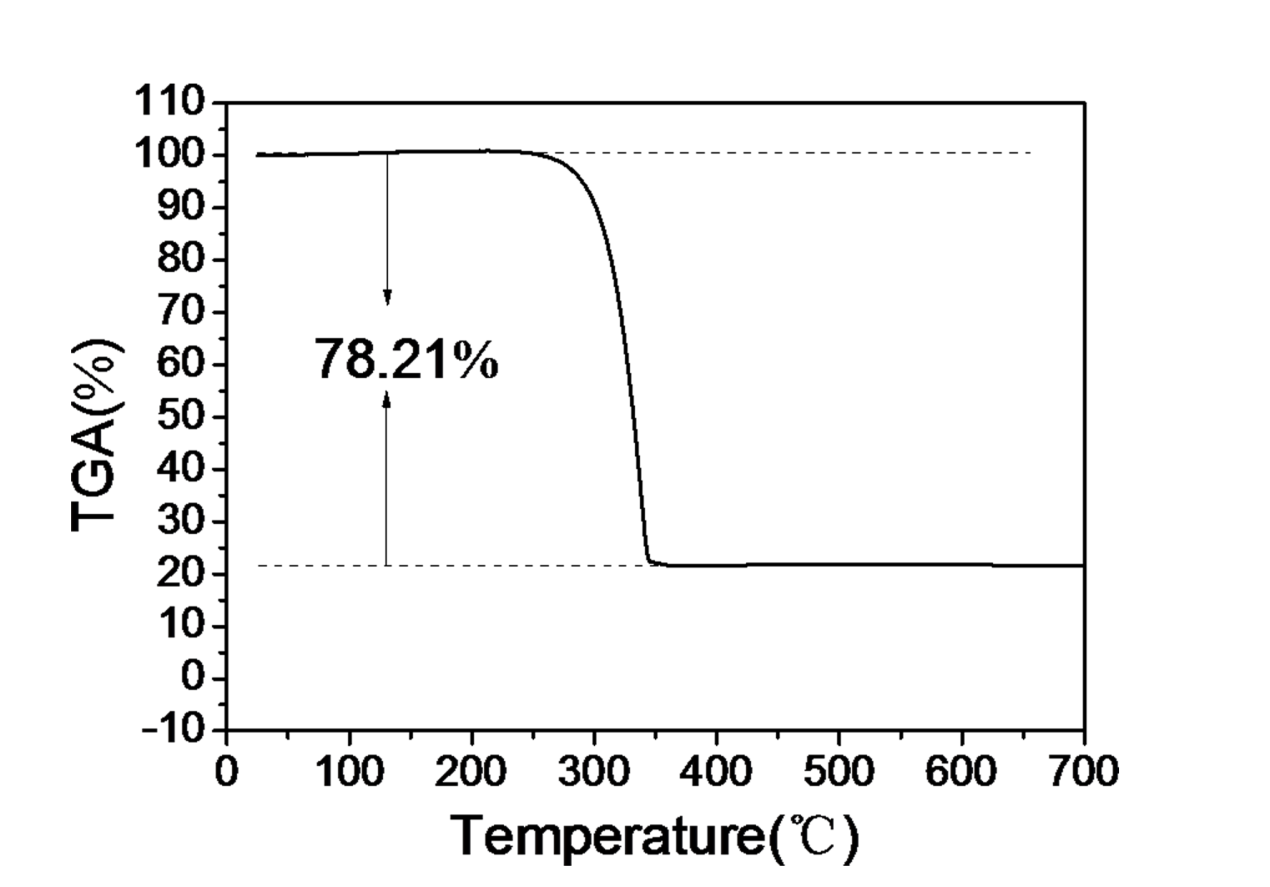


**Figure S2** The TGA result of Cd(DDTC)_2_.
